# Screening a mushroom extract library for activity against *Acinetobacter baumannii *and *Burkholderia cepacia *and the identification of a compound with anti-*Burkholderia *activity

**DOI:** 10.1186/1476-0711-9-4

**Published:** 2010-01-21

**Authors:** William R Schwan, Craig Dunek, Michael Gebhardt, Kathleen Engelbrecht, Tiffany Klett, Aaron Monte, Joseph Toce, Marc Rott, Thomas J Volk, John J LiPuma, Xue-Ting Liu, Ronald McKelvey

**Affiliations:** 1 Department of Microbiology, University of Wisconsin-La Crosse, La Crosse, WI, USA; 2 Department of Chemistry, University of Wisconsin-La Crosse, La Crosse, WI, USA; 3 Department of Biology, University of Wisconsin-La Crosse, La Crosse, WI, USA; 4 Department of Pediatrics, University of Michigan Medical School, Ann Arbor, MI, USA

## Abstract

**Background:**

*Acinetobacter baumannii *and species within the *Burkholderia cepacia *complex (BCC) are significant opportunistic bacterial pathogens of humans. These species exhibit a high degree of antibiotic resistance, and some clinical isolates are resistant to all currently available antimicrobial drugs used for treatment. Thus, new drugs are needed to treat infections by these species. Mushrooms could be a potential source for new drugs to treat *A. baumannii *and BCC infections.

**Methods:**

The aim of this study was to screen a library of crude extracts from 330 wild mushrooms by disk diffusion assays for antibacterial activity against *A. baumannii *and *Burkholderia cepacia *in the hope of identifying a novel natural drug that could be used to treat infections caused by these species. Once positive hits were identified, the extracts were subjected to bioassay-guided separations to isolate and identify the active drug molecules. MICs were performed to gauge the *in vitro *activity of the purified compounds.

**Results:**

Only three crude extracts (0.9%) had activity against *A. baumannii *and *B. cepacia*. Compounds from two of these extracts had MICs greater than 128 μg/ml, and further analyses were not performed. From the third extract, prepared from *Leucopaxillus albissimus*, 2-aminoquinoline (2-AQ) was isolated. This compound exhibited a modest MIC *in vitro *against strains from nine different BCC species, including multi-drug resistant clinical isolates (MIC = 8-64 μg/ml), and a weak MIC (128 μg/ml) against *A baumannii*. The IC_50 _against a murine monocyte line was 1.5 mg/ml.

**Conclusion:**

The small number of positive hits in this study suggests that finding a new drug from mushrooms to treat Gram-negative bacterial infections may be difficult. Although 2-AQ was identified in one mushroom, and it was shown to inhibit the growth of multi-drug resistant BCC isolates, the relatively high MICs (8-128 μg/ml) for both *A. baumannii *and BCC strains suggests that 2-AQ is not suitable for further drug development in its current form.

## Background

Glucose nonfermenting, Gram-negative rods (GNF-GNR) are commonly found in the environment and are typically responsible for opportunistic infections in severely ill and immunocompromised patients. Because this group of bacteria can survive in diverse environments, they are important opportunistic pathogens [1,2]. *Pseudomonas aeruginosa *is a leading cause of nosocomial infections due to GNF-GNR, followed by *Acinetobacter *spp., *Stenotrophomonas maltophilia*, and *Alcaligenes *spp. [3]. Although species in the *Burkholderia cepacia *complex (BCC) do not often cause human infections, it has been known for thirty years that infections that do occur are difficult to treat [4]. The BCC comprises a group of at least 17 species [5,6], although *B. cenocepacia *and *B. multivorans *are the most common species recovered from cystic fibrosis (CF) patients [4,7]. Besides CF patients, BCC also causes respiratory infections in hospitalized immunocompromised patients [8,9]. Some strains of the BCC are resistant to all known antibiotics, including the front line drugs, trimethoprim/sulfamethoxazole, piperacillin, ceftazidime, ciprofloxacin, and pipericillin-tazobactam [10,11].

For the GNF-GNR, multidrug resistance is becoming increasingly common [3,12]. This has led to the loss of most classes of antibiotics to treat infections caused by this group of bacteria, resulting in the use of polymyxins as the only remaining option to treat many of the infections [12]. Unfortunately, polymyxins used for systemic infections carry with them an increased risk of nephrotoxicity in the patient population being treated. Moreover, BCC strains are inherently resistant to polymyxins.

Small molecules synthesized randomly through combinatorial chemistry techniques have been suggested as one possible solution to bacterial drug resistance; however, these molecules generally lack the complexity of those derived from natural sources [13,14]. Historically, broad screening of natural products for antibacterial activity has yielded many active compounds, with several being derived from secondary metabolites of the mycelium of filamentous fungi [15]. In addition, because of their natural resistance to degradation by many bacteria in the soil, fungal fruiting bodies (i.e. mushrooms) remain an untapped reservoir of novel natural products. Moreover, it is estimated that only 5% of the approximately 1.5 million fungal species have been described and chemically characterized [16]. Thus, wild mushrooms represent a potentially rich, untapped source of new antimicrobial agents. In this study, we screened a library of crude extracts from mushrooms for activity against *A. baumannii *and *B. cepacia*, hoping to find a new natural drug that could be used to treat infections caused by multi-drug resistant strains of these species.

## Methods

### Construction of a mushroom crude extract library

Three hundred and thirty wild mushrooms were collected from across the United States. Most the mushrooms were collected in Minnesota and Wisconsin, although some were found in Colorado, Illinois, Michigan, New Jersey, North Carolina, Oregon, Pennsylvania, and Tennessee. A total of 59 different mushroom families were represented in this collection. The family *Tricholomataceae *was represented most often (26 mushrooms) followed by the family *Agaricaceae *(16 mushrooms). Mushroom sample weights from each site ranged from 10 g to 70 g, representing one to 25 fruiting bodies. Each mushroom was identified to the species level based on the structural characteristics of the fruiting body and air-dried in a commercially available food dehydrator. Five grams of each dried fungal material was extracted for 4 h at room temperature in the dark with 20 mL of a 5% methanol-95% dichloromethane solution. After filtration, the volatiles were removed by evaporation under a stream of N_2 _gas to yield the crude extract in a tared glass vial.

### Strains

The bacterial strains used in this study are listed in Table 1. All *Burkholderia *strains were identified to the species level at the *Burkholderia cepacia *Reference Laboratory and Repository (University of Michigan) by polyphasic analyses using phenotypic and genotypic assays as described previously [8]. All *Burkholderia *isolates also were subjected to repetitive extragenic element-PCR typing using the BOX A1R primer as previously described [17] to ensure that all isolates were genotypically distinct. Bacteria were stored at -80°C in skim milk or Luria-Bertani broth (Invitrogen) with 15% glycerol and recovered from frozen stock overnight at 37°C on Mueller-Hinton (MH; Difco) agar. Bacteria were grown on trypticase soy agar (BBL) overnight at 35°C before use in susceptibility testing.

**Table 1:** Bacteria used in this study

Bacteria	Strain	Source/reference^a^
*Achromobacter xylosoxidans*	MC	MC
*Achromobacter xylosoxidans*	ATCC 9220	ATCC
*Acinetobacter baumannii*	UWL	UWL
*Acinetobacter baumannii*	ATCC 19606	ATCC
*Burkholderia ambifaria*	AU4587	BcRLR
*Burkholderia ambifaria*	AU5203	BcRLR
*Burkholderia ambifaria*	AU11161	BcRLR
*Burkholderia cepacia*	ATCC 25416	ATCC
*Burkholderia cepacia*	AU0108	BcRLR
*Burkholderia cepacia*	AU2720	BcRLR
*Burkholderia cepacia*	AU2769	BcRLR
*Burkholderia cepacia*	AU7554	BcRLR
*Burkholderia cenocepacia*	J2315	BcRLR
*Burkholderia cenocepacia*	AU6550	BcRLR
*Burkholderia cenocepacia*	AU9292	BcRLR
*Burkholderia cenocepacia*	AU10321	BcRLR
*Burkholderia dolosa*	AU0794	BcRLR
*Burkholderia dolosa*	AU3271	BcRLR
*Burkholderia dolosa*	AU9336	BcRLR
*Burkholderia dolosa*	AU9628	BcRLR
*Burkholderia dolosa*	AU12872	BcRLR
*Burkholderia gladioli*	AU3431	BcRLR
*Burkholderia gladioli*	AU4123	BcRLR
*Burkholderia gladioli*	AU9405	BcRLR
*Burkholderia multivorans*	ATCC 27616	ATCC
*Burkholderia multivorans*	AU5573	BcRLR
*Burkholderia multivorans*	AU7455	BcRLR
*Burkholderia multivorans*	AU10398	BcRLR
*Burkholderia multivorans*	AU4507	BcRLR
*Burkholderia pyroccinia*	AU5468	BcRLR
*Burkholderia pyroccinia*	AU7314	BcRLR
*Burkholderia pyroccinia*	AU1114	BcRLR
*Burkholderia stabilis*	AU4757	BcRLR
*Burkholderia stabilis*	AU5832	BcRLR
*Burkholderia stabilis*	AU9035	BcRLR
*Burkholderia vietnamiensis*	AU3032	BcRLR
*Burkholderia vietnamiensis*	AU3578	BcRLR
*Burkholderia vietnamiensis*	AU3997	BcRLR
*Burkholderia vietnamiensis*	AU5003	BcRLR
*Burkholderia vietnamiensis*	AU10214	BcRLR
*Pseudomonas aeruginosa*	ATCC 27853	ATCC
*Stenotrophomonas maltophilia*	UWL	UWL
*Stenotrophomonas maltophilia*	ATCC 13637	ATCC

^a ^ATCC, American Type Culture Collection; BcRLR, *Burkholderia cepacia *Research Laboratory and Repository (University of Michigan);UWL, the University of Wisconsin-La Crosse Culture Collection; MC, Marshfield Clinic.

### Antimicrobial agents

The following antibiotics were used in Kirby Bauer disk diffusion and MIC assays: tetracycline, trimethoprim-sulfamethoxazole, tobramycin, gentamicin, amikacin, aztreonam, ceftazidime, meropenem, imipenem, ticaricillin/clavulonic acid, piperacillin/tazobactam, ciprofloxacin, piperacillin, cefepime, and levofloxacin. The antibiotics were purchased from Fluka or Sigma-Aldrich.

### Disk diffusion assays

All Kirby Bauer disk diffusion assays were performed according to Clinical and Laboratory Standards Institute (CLSI) guidelines [18]. Tetracycline and dimethylsulfoxide (DMSO) were used as controls in these assays. A zone of inhibition of growth greater than 6 mm was considered positive in this screening procedure.

### Bioassay-guided separation

For those extracts that showed a zone of inhibition of growth of either *A. baumannii *UWL or *B. cepacia *ATCC 25416, the extract was fractionated by successive thin-layer chromatography (TLC), using 500 mm thickness Whatman 20 cm × 20 cm, 60 Å silica gel plates with fluorescent indicator, eluting initially with 5% methanol-95% dichloromethane and then 50% isopropanol-50% acetonitrile. Following development of the TLC plates, one fraction with activity against these bacteria was visualized under short wave UV light (254 nm) as a relatively low *R*_f_, violet-colored band following development of the TLC plates. Final purification of this bioactive compound was accomplished by preparative scale high performance liquid chromatography (HPLC) using a Waters HPLC system with diode array detector, reverse phase C-18 column (Alltech), and a 1% ammonia-55% methanol-44% water solution as the eluant. The major component that eluted from the column retained the previously observed bioactivity, and this purified sample also was used for subsequent structure elucidation studies. Structural analyses were performed using positive ionization high-resolution mass spectrometry (HRMS; Varian Saturn mass spectrophotometer), proton and carbon NMR spectroscopy (500 MHz), and IR spectroscopy (thin film method).

### MIC assays

All MIC assays were performed according to CLSI regulations [18]. *B. cepacia *ATCC 25416 was used as a control for these assays. Each strain was tested a minimum of three separate times to ensure reproducibility. Commercially available 2-AQ (Sigma-Aldrich) was used in side-by-side comparison testing with the naturally derived 2-AQ and in the screening of several BCC strains.

### Cytotoxicity assays

To screen for cytotoxicity, two assays were used. The first assay used sheep blood agar plates and two-fold dilutions of the compound in phosphate buffered saline (PBS, pH 7.2). Eight microliters of the diluted drug were added to a 6 mm paper disk on a sheep blood agar plate. The plates were incubated for 16 to 18 h at 35°C. A zone of lysis was considered positive and the last dilution exhibiting a hemolysis zone was considered the IC_50 _for the drug.

The second assay utilized was a trypan exclusion assay [19]. Murine J774A.1 (ATCC) and human U937 (ATCC) monocyte cell lines were tested. Monocytes were seeded at 1 × 10^6^/well in a 24-well tissue culture plate with RPMI 1640 medium (Invitrogen). The next day, two-fold dilutions of the drug in PBS were added to each well with RPMI 1640 medium. On the following day, the spent medium was removed by aspiration and a trypan blue solution was added to each well for 10 min. After the 10 min elapsed, the trypan blue solution was aspirated and an equal volume of PBS was added. Three fields of cells were counted per well. The number of dead (blue) and live (clear) cells was determined. An IC_50 _was calculated for the well with 50% dead cells.

## Results and Discussion

### Screening the mushroom library

New drugs are needed to treat infections caused by Gram-negative bacteria. The Antimicrobial Availability Task Force (AATF) of the Infectious Diseases Society of America (IDSA) has listed several microorganisms that are viewed as significant health problems [20]. Among the species listed are the GNF-GNR species *P. aeruginosa *and *A. baumannii*, predominantly because of the antibiotic resistance profiles for both species and the dearth of potentially effective drugs in the late-stage development pipeline. Moreover, extensively drug resistant *A. baumannii *that are even resistant to colistin are associated with high mortality rates [21]. In general, the pipeline for viable/strong candidate antibacterial drugs is lacking [22-24]. Currently, there are no new drugs with novel mechanisms of action in Phase I clinical studies to treat Gram-negative bacterial infections [25]. Furthermore, there are very few preclinical leads that exhibit favorable profiles against Gram-negative bacteria.

In an attempt to find new drugs to treat infections caused by GNF-GNR, 330 wild mushroom crude extracts were tested for their activity against *A. baumannii *UWL and *B. cepacia *ATCC 25416, but only three extracts showed a zone of inhibition of growth greater than 6 mm against both strains. The DMSO controls displayed no zone of inhibition (reported as 6 mm), whereas tetracycline displayed a large inhibition zone against both species. In the subsequent screening of the three crude extracts, an extract from the *Leucopaxillus albissimus *mushroom was identified as the only one showing promising activity against either *A. baumannii *UWL or *B. cepacia *ATCC 25416 The zone of antibacterial activity of this extract was larger against *B. cepacia *compared to the zone for *A. baumannii*.

### Purification of the active compound from an *L. albissimus* crude extract

The major active component from *L. albissimus *was determined to be 2-aminoquinoline (2-AQ), also known as 2-quinolylamine, on the basis of its spectral and physical data. Its positive ionization high-resolution mass spectrum (HRMS) showed an exact mass of 145.07573 g/mol for the M+1 ion, indicating a molecular formula of C_9_H_8_N_2 _consistent with that of 2-AQ. The proton and carbon NMR spectra were essentially identical to those reported in the literature for 2-AQ [26]. The IR spectrum (thin film method) of the natural product was superimposable on the IR spectrum for 2-AQ contained in the online SDBS spectral database. Finally, the HPLC retention time and NMR spectra of the purified natural product were compared with those of an authentic synthetic sample obtained through Sigma-Aldrich, and these were identical, as were the biological activities of the two samples.

### Antibacterial activity assessment of 2-AQ

Although antibacterial activity was previously attributed to 2-AQ [26,27], GNF-GNR species, such as BCC, *P. aeruginosa*, *A. xylosoxidans*, *A. baumannii*, and *S. maltophilia*, were not tested in earlier studies. To determine the *in vitro *activity of the 2-AQ natural product, a MIC analysis was performed to quantitatively measure its activity against BCC species and other GNF-GNR [13]. The MIC of 2-AQ for *B. cepacia *ATCC 25416 was 32 μg/ml, but it was 16 μg/ml for *B. cenocepacia *J2315 and *B. multivorans *ATCC 27616. The MIC of 2-AQ was 64 μg/ml for *S*. *maltophilia *ATCC 13637, 32 μg/ml for *A. xylosoxidans *ATCC 9220, and 128 μg/ml for both *P. aeruginosa *ATCC 27853 and *A. baumannii *ATCC 19607 (TABLE 2). No inhibitory activity of 2-AQ was observed for *Escherichia coli, Staphylococcus aureus*, or *Enterococcus faecalis *(data not shown). Commercially available 2-AQ was also tested side-by-side with the natural product against the same species. No MIC difference was observed comparing the natural product to the commercially prepared sample. The same bacterial strains were tested against tetracycline, a drug class that has been proven effective against a wide variety of GNF-GNR bacteria, and the results are also shown in TABLE 2. Although tetracycline was more active against some of the bacteria tested, the tetracycline MIC was equivalent against *B. cepacia *ATCC 25416 (32 μg/ml), two- to four-fold higher against the two *A. xylosoxidans *strains (128 μg/ml), and four-fold higher against *B. cenocepacia *J2315 when compared to the 2-AQ MIC (64 mg/ml). In addition, the MICs of piperacillin and ceftazidime against the ATCC strains of *B. cepacia *and *P. aeruginosa *were both 1 μg/ml. Moreover, the MIC of sulfamethoxazole-trimethoprim (SXT) against *B. cepacia *ATCC 25416 was 0.5 μg/ml.

**Table 2:** MICs of 2-aminoquinoline (2-AQ) isolated from *L. albissimus *and tetracycline (Tc) against several bacterial species

Species	Strain	MIC^a^	
		2-AQ^b^	Tc^b^
*Pseudomonas aeruginosa*	ATCC 27853	128	8
*Burkholderia cepacia*	ATCC 25416	32	32
*Burkholderia cenocepacia*	J2315	16	64
*Burkholderia multivorans*	ATCC 27616	16	2
*Stenotrophomonas maltophilia*	UWL	32	0.25
*Stenotrophomonas maltophilia*	ATCC 13637	64	1
*Achromobacter xylosoxidans*	MC	64	128
*Achromobacter xylosoxidans*	ATCC	32	128
*Acinetobacter baumannii*	UWL	128	0.5
*Acinetobacter baumannii*	ATCC 19606	128	2

^a ^MIC given in μg/ml.

^b ^2-AQ,2-aminoquinoline; Tc, tetracycline.

To determine if 2-AQ had broad anti-*Burkholderia *activity, a diverse panel of BCC strains as well as several *Burkholderia gladioli *strains were tested using the commercially prepared 2-AQ. These strains represented *B. gladioli *and eight species within the BCC. Some of the strains were environmental isolates, whereas others were multi-drug and pan-drug resistant strains collected from hospitalized patients. These results showed that there was *in vitro *activity against *B. gladioli *and all of the BCC strains (MICs of 16 to 64 μg/ml) (TABLE 3). Of the species assayed, the five *B. cepacia *strains had the lowest MIC range (8-32 μg/ml), whereas the *B. stabilis *had the highest MICs (64 mg/ml). Multi-drug resistant strains of *B. ambifaria *(AU5203 and AU11161), *B. cepacia *(AU0108), *B. cenocepacia *(all strains except J2313), *B. dolosa *(AU0936 and AU12872), *B. multivorans *(AU4507, AU5573, AU7455, and AU10398), *B. pyroccinia *(AU7324 and AU1114), *B. stabilis *(AU9035) and *B. vietnamiensis *(AU10214) were examined in this study. They all had 2-AQ susceptibility patterns similar to non-multi-drug resistant environmental isolates. Although *B. gladioli *is not part of the BCC, the strains that were tested also had high MICs (64 mg/ml). All of the *B. cenocepacia *clinical isolates tested either displayed full resistance to all antibacterial drugs (AU10321) or intermediate and full resistance (AU9292 and AU6550) to the same panel of 13 drugs that included trimethoprim/sulfamethoxazole, tobramycin, gentamicin, amikacin, aztreonam, piperacillin, piperacillin/tazobactam, ticaricillin/clavulonic acid, ceftazidime, cefepime, meropenem, imipenem, and levofloxacin (TABLE 4) [28]. The *in vitro *activity of 2-AQ suggests that the molecule has weak activity against bacterial pathogens that affect CF patients, including highly drug resistant strains of *B. cepacia *complex.

**Table 3:** MIC range of commercially available 2-AQ activities against *Burkholderia *strains

Species (μg/ml)	Strains Tested	MIC Range
*B. ambifaria*	3	16-64
*B. cenocepacia*	4	16-64
*B. cepacia*	5	8-32
*B. dolosa*	5	32-64
*B. gladioli*	3	64
*B. multivorans*	5	32
*B. pyroccinia*	3	32-64
*B. stabilis*	3	64
*B. vietnamiensis*	5	32-64

**Table 4:** Comparison of commercially prepared 2-AQ MICs versus frontline drug MICs against multidrug-resistant *B. cepacia *complex clinical isolates

					Antibacterial drug MIC (μg/ml)									
Bacterial strain	SXT^a^	NN	GM	AN	ATM	PIP	TZP	TIC	CAZ	FEP	MEM	IPM	LVX	2-AQ
*B. multivorans *AU5573	0.25	256	64	64	16	32	8	128	8	8	4	32	1	64
*B. multivorans *AU7455	1	64	>64	>64	16	8	8	32	8	2	2	32	2	32
*B. multivorans *AU10398	1	>256	>64	>64	32	-^b^	32	>128	>64	>32	8	16	16	16
*B. cenocepacia *AU6550	4	>256	>64	>64	>64	-	128	>128	16	>32	16	>32	16	16
*B. cenocepacia *AU9292	>4	>256	>64	>64	>64	>128	>128	>128	32	>32	16	>32	>16	64
*B. cenocepacia *AU10321	4	>256	>64	>64	>64	>64	>128	>128	64	>32	32	>32	>16	32

^a^SXT = trimethoprim/sulfa, NN = tobramycin, GM = gentamicin, AN = amikacin, ATM = aztreonam, PIP = piperacillin, TZP = piperacillin/tazobactam, TIC = ticaricillin/clavulanic acid,, CAZ = ceftazidime, FEP = cefepime, MEM = meropenem, IPM = imipenem, LVX = levofloxacin, and 2-AQ = 2-aminoquinoline.

^b ^- = not determined.

### *In vitro *cytotoxicity testing of 2-AQ

A crude cytotoxicity test using sheep red blood cells demonstrated that cell lysis occurred when the concentration of 2-AQ was ≥ 1.6 mg/ml, suggesting that the drug might have a low cytotoxic effect. Furthermore, trypan blue exclusion experiments using 2-AQ against J774A.1 and U937 murine monocytic cell lines demonstrated that it took 1.5 mg/ml of 2-AQ to injure 50% of the eukaryotic cells and 500 μg/ml to injure either cell line. These results demonstrate that 2-AQ has a cytotoxicity profile against mammalian cells only 10 to 30-fold higher than its MICs against GNF-GNR.

In this study, 2-AQ was identified as a natural product that targets GNF-GNR, particularly the BCC. With the number of multi-drug resistant BCC strains on the rise [12,28,29], a new drug is sorely needed, particularly to treat BCC strains that display resistance to the frontline antibacterial drugs currently in use [29,30]. The *in vitro *MIC values for 2-AQ were the lowest against the *B. cepacia *strains within the BCC; however, they were still too high to be considered for further commercial development of 2-AQ in its current form. Furthermore, 2-AQ is commercially available and is considered to be a toxic compound. It is possible that an analog of 2-AQ could be developed with a more favorable therapeutic index. Presently, 2-AQ analogs are being developed as melanin-concentrating hormone antagonists [31,32].

## Conclusion

Screening of a library of wild mushroom extracts for antibacterial activity against *A. baumannii *and *B. cepacia *yielded limited results. Only three mushroom extracts displayed any activity, and further investigation of the mushroom with the most promising activity, *L. albissimus*, resulted in the isolation of a known compound, 2-AQ, that had modest activity against several BCC strains and limited potential for commercial development.

## Competing interests

WRS, AM, MR, and TV have filed a patent on 2-AQ.

## Authors' contributions

WRS performed laboratory studies, drafted the manuscript, and edited the manuscript; AM, MR, KE, and TV created the mushroom library and edited the manuscript; MG, KE, CD, and TK screened the mushroom library and assisted with the purification of the active drug; JT performed the HPLC separation of the purified 2-AQ compound; JJL provided the clinical isolates, ran the MICs on the clinical isolates, and edited the manuscript; and RM and XTL obtained and interpreted the MS, IR, and NMR spectra. All authors read and approved the final manuscript.
